# Different effects of rumination on depression: key role of hope

**DOI:** 10.1186/1752-4458-8-53

**Published:** 2014-12-13

**Authors:** Haitao Sun, Qinyi Tan, Guanhua Fan, Qien Tsui

**Affiliations:** School of Law, Hohai University, Nanjing, China; Center for Studies of Education and Psychology of Ethnic Minorities in Southwest China, Southwest Universitiy, Chongqing, China; Lab of Human Resource Assessment and Development, Nankai University, Tianjin, China; Educational Research Center of Western Guangdong Province, Lingnan Normal University, Zhanjiang, China

**Keywords:** Rumination, Hope, Depression, Moderating effect

## Abstract

**Background:**

Both rumination and hope have significant impacts on depression. However, few studies concern their trilateral relationship. This study examined the moderator effect of hope on the relationship between rumination on depression in Chinese university students.

**Methods:**

517 college students completed the measures of rumination, hope and depression.

**Results:**

Hierarchical regression analysis showed that hope moderated the association between rumination and depression. When students reported a low level of hope, those with high rumination reported higher scores in depression than those with low rumination. However, in high hope group, the effect of rumination on depression became not significant.

**Conclusions:**

Hope can significantly moderate the effect of rumination on depression. The significance and limitations of the results are discussed.

## Introduction

Rumination is a type of negative reaction; it means that when an individual is in pain, he or she dwells on such emotion and its causes and various results instead of taking active measures to solve the problem [1, 2]. Several researchers regard rumination as a personal trait reflected by the condition where an individual overthinks pain and immerses himself in the circumstance to limit his motivation to communicate and curb his active behavior [3–5]. Individuals who are used to dealing with negative events through such reaction means, namely, rumination, dwell on the negative emotion although the matter is over and things have changed [6].

Nolen-Hoeksemaetc believed that the origin of rumination is related to childhood experience [7]. If an individual was unable to learn active emotion management in his early childhood, he would be likely to exhibit rumination after overstimulation from parents or sex abuse and would control on the environment. When parents exhibit negative behavior in life, they provide an example to their children, who would eventually exhibit rumination [8, 9]. The origin of rumination is also related to personal character; for example, individuals with high pursuit for perfectionism, high social anxiety, pessimism, and a neurotic character are highly likely to perform rumination [10, 11]. Several scholars, such as Watkins, explained the formation mechanism of rumination based on reduced concreteness theory [12]. According to the theory, individual thinking are of two types, namely, concrete and abstract. Concrete thinking is characterized by cross-circumstance and ambiguity, such as abstract autobiography memory (“I am always a loser” or “I always do something wrong”), which is the conclusion of repeated experience. Rumination is usually accompanied by abstract autobiography memory. Thus, the negative effect caused by concrete thinking influences problem processing and solving and may eventually cause psychological discomfort [12, 13].

Many studies have reported that rumination is related to negative effects [14, 15]. Nolen-Hoeksema et al. studied and monitored depression in adults who have lost their relatives. They found that rumination can still predict the depression level for the next six months if the variation, including depression level, social support, pressure source, and gender, is controlled [16, 17]. Lyubomirsky and Lepper found that depression worsens when rumination induced by experiments is increased [18]. According to a longitudinal research, an individual immersed in negative emotions and tends to ruminate is likely to suffer from serious depression, with more instances and longer continuance [19, 20]. An individual who is healthy but has the tendency to ruminate could easily become depressed. Rumination aggravates the illness of clinical patients with depression [21]. Similar research results have also been obtained on the other negative effects of rumination, including anxiety and anger [22]. Rumination has also been found to be related to clinical mental illnesses (e.g., suicidal ideation and post-traumatic stress) [23, 24]. Researchers believe that when an individual faces traumatic life events and experiences negative emotions (e.g., depression), he constantly thinks about the causes and results of negative emotions. This overthinking activates the previous negative memory and results in a negative reaction to the present circumstance. The sense of failure and helplessness is then strengthened. Therefore, under the influence of rumination, the social function of individual psychology may induce a morbid state, which would eventually develop into emotional disorders, including depression and anxiety [25].

As an important positive psychological trait, hope has elicited much attention from scholars; it is regarded as a protective factor by people faced with risks [26]. Snyder defined hope as an internal sense of success and an active motivation state [27]; compared with an individual with a low level of hope, an individual with a high hope level is more firm and flexible and seeks alternative means to realize his expected goal when faced with failure to mitigate the negative effect induced by negative events [27]. Previous research has indicated that hope plays a protective role in depression and has a negative correlation with depression [28]. The current study explores the relations between rumination and depression in undergraduates and the moderating effect of hope on depression.

## Methods

### Participants and procedure

Participants were 517 undergraduates from two general universities in China, which consisted of 224 women and 293 men. The ages of participants ranged from 19 to 23, with a mean of 20.58 (SD = 1.44), all unmarried. Participants received ¥15 as compensation. 517 scales were distributed and collected, and all were valid. All participants provided their written informed consent before completing the measures.

### Instruments

#### Ruminative response scale

The Ruminative Response Scale (RRS) is designed to assess ruminative coping responses to depressed mood. The RRS has been shown to have good internal consistency and validity [7]. The scale contains 22 items, and the responses to each item are scored from 1 (‘almost never’) to 4(‘almost always’). The scores on all of the items are added to obtain the total score, which ranges from 22 to 88, with higher scores indicating higher levels of ruminative coping responses. RRS was translated into Chinese by Hong and his colleges, and has been proven to have good validity and reliability for the Chinese population [29]. In the present study, the Cronbach alpha coefficient for the RRS was 0.71.

### Hope scale

Hope was measured using the Hope Scale, a 12-item self-report measure [27]. The scale consists of two subscales: agency and pathways. The pathways items measure the ability to develop goal achieving strategies. The agency items assess the extent to which a person believes that they can achieve their goals through their pathways. The Hope Scale has shown good test–retest reliability and high internal consistency has been observed for both subscales [30]. Hope scale was translated by Zhang, Gao, Wang and Wu in the year of 2010, and has been proven to have good validity and reliability [31]. In the current study, the Cronbach alpha coefficient for the Hope scale was 0.77.

### Self-rating depression scale (SDS)

Self-rating Depression Scale (SDS), developed by Zung, is a self-report measure of depression consisting of 20 items, with a four-point scale ranging from a little of the time (1) to most of the time (4). Of the 20 items, 10 are worded positively and 10 are worded negatively. The former 10 items are reversed items. The validity and the reliability of the SDS have been reported [32]. The researcher translated the 20-item version of SDS into Chinese, and the Chinese vision of the scale has been proved to have good validity and reliability [33]. In this study, the Cronbach alpha coefficient for SDS was 0.82.

## Results

### Bivariate analyses

Means, standard deviations, and intercorrelations for each questionnaire are presented in Table 1. Rumination was negatively correlated with hope (r = -0.52, p < 0.01). In addition, hope was negatively correlated with depression (r = -0.42, p < 0.01), and rumination was positively correlated with depression (r = 0.48, p < 0.01).

**Table 1:** **Means, standard deviations, and correlations of rumination, hope and depression**

	Mean	SD	1	2	3
1. Rumination	61.67	7.89	1		
2. Hope	32.73	3.71	-0.52^**^	1	
3. Depression	58.08	10.90	0.48^**^	-0.42^**^	1

Note: _**_, p < 0.01.

### Test of the moderation model of hope

In order to test the moderating effect of hope on the relationship between rumination and depression, hierarchical regression procedures were performed [34]. Before testing for moderating effect, the hope and rumination variables were standardized to reduce problems related to multicollinearity between the interaction term and the main effects [35]. Thus, z-scores were calculated for hope and rumination. In the hierarchical regression model, the order of entry was as follows. At Step 1, the predictor variable (rumination) was entered into the regression equation. At Step 2, the moderator variable (hope) was entered into the regression equation. At Step 3, the interaction of rumination × hope was added. Significant change in R^2^ for the interaction term indicates a significant moderator effect. Results of these analyses are presented in Table 2. As indicated in Table 2, control variables were not able to enter in the first step. Rumination (β = 0.47, p < 0.01) predicted significantly to depression in the second step. The hope factor added a significant increment to the model in step three, where hope (β = -0.19, p < 0.01) was significantly related to depression after controlled for the other variables. Additionally, as predicted, there was a significant interaction between rumination and hope (β = -0.12, p < 0.01). These findings suggest that hope moderated the impact of rumination on depression.

**Figure 1:**
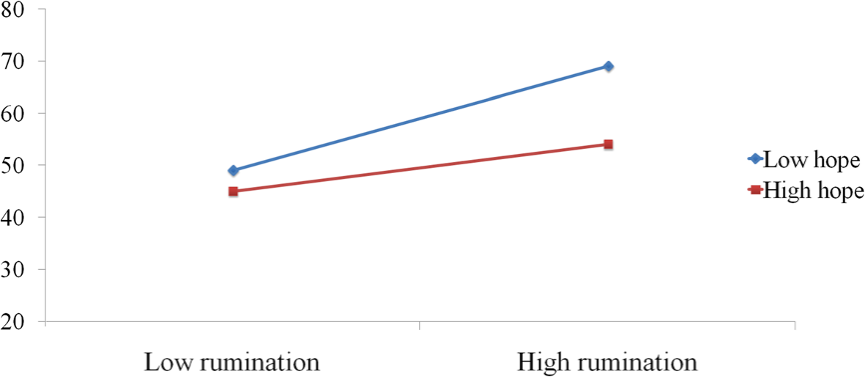
**The moderate role of hope in the relation between rumination and depression**.

To illustrate the rumination × hope interaction for depression, we plotted the regression of depression on rumination at high and low levels of hope. Consistent with procedures outlined by Peng and his colleges, we used the simple slope for the regression of rumination on depression. Those whose rumination scores were ±1 Z score away from the sample mean were considered as high and low rumination individuals. The results suggested that, there was a significant positive relation between rumination and depression at low levels of hope (β = 0.70, p < 0.01). However, at high levels of hope, the relation between rumination and depression was not significant (β = 0.05, p = 0.67), see Figure 1. Hence, the results also suggest that hope moderated the impact of rumination on depression.

**Table 2:** **Hierarchical regression analysis predicting depression from rumination and hope**

	***β***	***t***	***F***	R ^2^	△R ^2^
Step 1					
Rumination	0.48	12.34	152.19	0.228	0.228
Step 2					
Hope	-0.24	-5.44	29.64	0.270	0.042
Step 3					
Rumination × Hope	-0.21	-5.73	32.87	0.314	0.044

## Discussion

The results of this research indicated that rumination is a cognitive predisposing factor of depression and may aggravate the correlation between depression and negative cognition; this finding is consistent with that in previous studies [19, 36]. College students encounter many negative life events, including academic, interpersonal communication, vocational development, and social adaption problems. They cannot escape from the effect of these negative events and repeatedly think “why did these things happen to me” or “how sad I feel”. Negative events continuously harm an individual’s psychological health and may cause or aggravate depression. An individual with high rumination constantly ponders an issue with negative cognition means, enlarges the negative consequence, complains consistently, and even lowers his or her self-evaluation; all of these further strengthen depression [37, 38].

This research mainly proves that hope plays a moderating role between rumination and depression. Specifically, for individuals with high hope, even a high level of rumination does not aggravate depression; the relation between rumination and depression is not significant. On the contrary, for individuals with low hope, high rumination means strong depression; specifically, rumination significantly aggravates depression. Such phenomenon may be explained from the following two perspectives. First, the ability of an individual to deal with negative events is determined by his or her level of hope. According to a number of studies, an individual with a high hope level has more self-respect, higher self-efficacy, and better behavior than an individual with a low hope level; the latter easily experiences depression or exhibits external behavior problems [39]. As a positive psychological capital, hope may provide a dynamic resource for an individual to effectively deal with risky events so as to reduce the ill effects brought about by risky events [40]. Second, individuals with different levels of hope may adopt different means of rumination. Several scholars believe that rumination has two dimensions, namely, forced thinking and introspection. These dimensions have different functions. Forced thinking may result in the occurrence of depression, extend the time of depression, and aggravate the degree of depression; thus, it is maladaptive. Meanwhile, introspection, in the long term, helps solve problems; hence, it is adaptive [41]. Considering that hope is a strong psychological capital, it may change the properties of the two dimensions of rumination. Specifically, rumination may have an active and adaptive function in individuals with high hope. Even if one performs maladaptive forced thinking (e.g., “why can’t I do the work better”), he or she will make full use of negative thoughts in an active manner to produce stronger motivation and seek better solutions to overcome the problem. On the contrary, rumination may have a negative and maladaptive function in individuals with low hope. For example, even if individuals with low hope employ adoptive means of introspection (e.g., “I will write down my own thoughts and analyze them”), they will explain the present conditions in a negative manner. They may think that they are too weak to solve the problem and could eventually give up. Therefore, hope plays an important role in determining whether rumination is adaptive or maladaptive.

The research also discussed the relations among rumination, hope, and depression, and meaningful conclusions were obtained. However, the research has several limitations. First, accurate causal relations were not obtained by the questionnaire employed in the research. Morrow et al. conducted experiments to induce rumination. The experimental method is recommended for use in future research to explore the effect of rumination on depression. Second, all the scales adopted in the current research are designed for western culture. Chinese and western culture are different, so cultural differences should be considered. Therefore, in future studies, the method of cross culture is recommended for the exploration of the negative effects of rumination.
